# Ion-exchange-resin-catalyzed adamantylation of phenol derivatives with adamantanols: Developing a clean process for synthesis of 2-(1-adamantyl)-4-bromophenol, a key intermediate of adapalene

**DOI:** 10.3762/bjoc.8.23

**Published:** 2012-02-08

**Authors:** Nan Wang, Ronghua Wang, Xia Shi, Gang Zou

**Affiliations:** 1 Department of Fine Chemicals, East China University of Science & Technology, Meilong Rd. 130, Shanghai, 200237, China. Fax: 86-21-64253881; Tel: 86-21-64252390

**Keywords:** adamantylation, ion-exchange resin, phenol, recycling

## Abstract

A clean process has been developed for the synthesis of 2-adamantylphenol derivatives through adamantylation of substituted phenols with adamantanols catalyzed by commercially available and recyclable ion-exchange sulfonic acid resin in acetic acid. The sole byproduct of the adamantylation reaction, namely water, could be converted into the solvent acetic acid by addition of a slight excess of acetic anhydride during the work-up procedure, making the process waste-free except for regeneration of the ion-exchange resin, and facilitating the recycling of the resin catalyst. The ion-exchange sulfonic acid resin catalyst could be readily recycled by filtration and directly reused at least ten times without a significant loss of activity. The key intermediate of adapalene, 2-(1-adamantyl)-4-bromophenol, could be produced by means of this waste-free process.

## Introduction

*o*-Adamantylphenols and their derivatives are the key skeletons of synthetic retinoid analogues [1–9] and supporting ligands of many homogeneous transition-metal catalysts [10–14]. Introduction of an adamantyl group to the phenol ring has largely relied on acid-catalyzed Friedel–Crafts alkylation with haloadamantanes or adamantanols [1–9,15–19]. Compared with adamantylation by haloadamantanes, the alkylation employing adamantanols combined with mineral acids, such as concentrated H_2_SO_4_, ensures that the process affords adamantylphenols under milder conditions, and thus often with higher selectivities. However, the work-up procedure of mineral-acid-promoted reactions necessitates aqueous quenching and neutralization steps to remove the acids, resulting in enormous quantities of waste. For example, although the state-of-the-art process promoted by combination of concentrated H_2_SO_4_ and glacial acetic acid in CH_2_Cl_2_ was adapted [20–22], it was still found that at least 15 kg of waste water, besides solid waste such as Na_2_SO_4_ and NaOAc, was generated per kilogram of 2-(1-adamantyl)-4-bromophenol, simply in an effort to remove the acids from the reaction mixture by means of aqueous quenching and washing. Therefore, the development of alternative procedures that can reduce or eliminate the waste from the work-up procedure is highly needed. It was reported that adamantylation of aromatics promoted by strong organic acids, such as trifluoroacetic acid [18] and triflic acid [19], could circumvent the drawbacks associated with using concentrated H_2_SO_4_. However, these strong organic protic acids are expensive and volatile, and thus their recovery poses many difficulties.

Strongly acidic ion-exchange sulfonic acid resins have been extensively exploited as recyclable acid catalysts for organic syntheses in both laboratory and industry since they became commercially available [23–26]. The success of recyclable ion-exchange resins as substitutes for mineral acids in organic syntheses prompted us to study the possibility of using acidic sulfonic acid resin as a recyclable catalyst in the adamantylation reaction of 4-bromophenol (**1a**) with 1-adamantanol (**2a**) to develop a clean process for the production of the key intermediate of adapalene, namely 2-(1-adamantyl)-4-bromophenol (**3aa**). Olah et al. [15] reported that amberlyst and nafion-H could effectively catalyze adamantylation of aromatics with 1-bromoadamantane similarly to organic sulfonic acids or their fluorinated analogues. However, due to the detrimental effects of water on the catalytic activity of the cation-exchange resin in Friedel–Crafts alkylation, there is, to the best of our knowledge, no report on cation-exchange-resin-catalyzed adamantylation of phenols with adamantanols, although alkylation with alcohols is greener than that with alkyl halides. Herein, we report a process catalyzed by a recyclable acidic resin for an efficient and clean adamantylation of phenol derivatives by using adamantanols [27].

## Results and Discussion

Treatment of 4-bromophenol (**1a**) with 1.05 equiv of 1-adamantanol (**2a**) in the presence of a macroporous sulfonic acid cation-exchange resin (Amberlite 200, H^+^ form) in ethyl acetate or diethyl carbonate under reflux for 10–12 h gave the desired product 2-(1-adamantyl)-4-bromophenol (**3aa**) in good yields; however, no reaction occurred in acetonitrile under similar conditions (Table 1, entries 1–3). The reaction proceeded faster in 1,2-dichloroethane but with lower selectivity, generating some (9%) doubly adamantylated product, 2,6-bis(1-adamantyl)-4-bromophenol, (Table 1, entry 4).

**Table 1:** Ion-exchange-resin-catalyzed adamantylation of 4-bromophenol (**1a**) with 1-adamantanol (**2a**).^a^

entry	resin loading (g)^b^	solvent	*T* (°C)	*t* (h)	yield (%)^c^

1	1.0	CH_3_CO_2_Et	reflux	12	94
2	1.0	CO(OEt)_2_	reflux	10	96
3	1.0	CH_3_CN	reflux	12	trace
4	1.0	(CH_2_Cl)_2_	reflux	5	83
5	1.0, 1^st^ recycle^d^	CO(OEt)_2_	reflux	24	76^e^
6	1.0, 2^nd^ recycle^f^	CO(OEt)_2_	reflux	15	93
7	1.0, 3^rd^ recycle^f^	CO(OEt)_2_	reflux	15	89
8	1.0	EtOH	reflux	12	trace
9	1.0	CH_3_CO_2_H	100	2	98
10	1.0	CH_3_CO_2_H	60	12	75
11	1.0	CH_3_CO_2_H	80	7	95
12	0	CH_3_CO_2_H	100	6	trace
13	0.30	CH_3_CO_2_H	100	6	92
14	0.75	CH_3_CO_2_H	100	4	98
15	0.75^g^	CH_3_CO_2_H	100	18	85

^a^The reaction was run at 3 mmol scale with respect to 4-bromophenol in air. ^b^Amberlite 200, H^+^ form used. ^c^Isolated yields. ^d^The recovered resin catalyst was dried in vacuum at 50 °C for 1 h. ^e^14% 4-bromophenol recovered. ^f^ The recovered resin catalyst was dried in vacuum at 90 °C for 3 h. ^g^Amberlite IR120 used instead of Amberlite 200.

The cation-exchange-resin catalyst could be easily recovered by filtration and washing with diethyl carbonate. However, the catalytic activity of the recovered resin catalyst strongly depended on the dryness of the resin. That is, the water byproduct had to be thoroughly removed from the recovered resin by drying the resin under vacuum (<0.5 mmHg) at 90 °C for 3 h before reuse, otherwise the reaction could not be carried to completion even after heating under reflux for 24 h (Table 1, entries 5–7). To facilitate recycling of the resin catalyst, we tested the reaction in ethanol, which was supposed to help in removing the byproduct water from the resin, but no reaction was observed after 12 h under reflux. However, when the reaction was conducted in acetic acid at 100 °C (bath temperature), almost a quantitative yield of the target product 2-(1-adamantyl)-4-bromophenol (**3aa**) was obtained (98% after purification by chromatography) in 2 h (Table 1, entry 9).

The control experiment showed that no reaction occurred in the absence of the acidic ion-exchange resin, clearly excluding the possibility of acetic acid catalysis in the adamantylation (Table 1, entry 12). When the loading of the resin catalyst was reduced to 0.3 g and 0.75 g from 1.0 g, for 3 mmol 4-bromophenol (**1a**), the adamantylation reaction still gave 92% and 98% yields of the target product, respectively, within 4–6 h (Table 1, entries 13 and 14). However, the microporous sulfonic acid cation-exchange resin (Amberlite IR120, H^+^ form) was found to be less effective in promoting the adamantylation compared to the macroporous analogue. For example, the reaction took a longer time and still gave a lower yield with Amberlite IR120 (Table 1, entry 15).

Acetic acid appeared to be the best choice of solvent for the resin-catalyzed adamantylation with 1-adamantanol (**2a**), as it not only gave the desired 2-(1-adamantyl)-4-bromophenol (**3aa**) in high yields within a shorter reaction time, but it also made it possible to directly reuse the recovered resin catalyst since the byproduct, water, which is detrimental to the catalytic activity of such resins, could be easily converted into acetic acid by the addition of an equivalent amount of acetic anhydride during the work-up procedure. Further, after the sole byproduct, water, is converted into the solvent acetic acid, the resulting process is waste-free except for regeneration of the resin at its first use. Therefore, both the resin catalyst and the acetic acid solvent can be easily recovered by simple filtration and readily reused.

**Figure 1:**
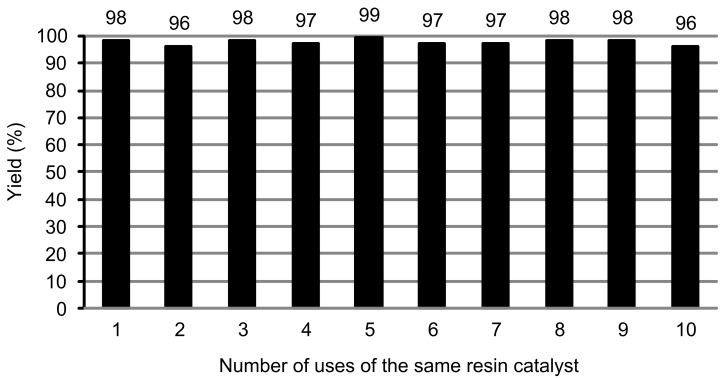
Recycling of the ion-exchange-resin catalyst in the adamantylation reaction of 4-bromophenol (**1a**) with 1-adamantanol (**2a**).

The recyclability of the acidic ion-exchange-resin catalyst was then investigated (Figure 1). To facilitate the operation, the reaction was run at 15 mmol scale with respect to **1a**, with 5.0 g of the resin catalyst. When the reaction was completed, the mixture was cooled to 50–60 °C, and an acetic acid solution containing 1.05 equiv acetic anhydride was added to the mixture in order to convert the byproduct, water, to the solvent, acetic acid, thereby realizing a waste-free process and removal of water from the resin in order to facilitate recycling of the resin catalyst. After being stirred for a while (1–2 h) to ensure complete consumption of water in the mixture, the resin catalyst was filtered, washed with acetic acid and used directly in the subsequent run. In fact, the resin catalyst, Amberlite 200, H^+^ form, was reused ten times without significant loss of activity in the reaction of 4-bromophenol (**1a**) with 1-adamantanol (**2a**) in acetic acid. Solvent acetic acid was recovered from the filtrate by distillation, and the residue, crude product 2-(1-adamantyl)-4-bromophenol (**3aa**), was further purified by recrystallization in CH_2_Cl_2_/petroleum ether.

The scope of the sulfonic acid resin catalyzed adamantylation of phenol derivatives with adamantanols was further explored (Table 2).

**Table 2:** Scope of the ion-exchange-resin catalyzed adamantylation.

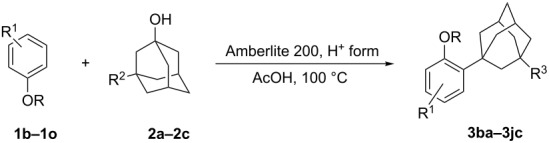					

entry	R/R^1^	R^2^	*t* (h)	R^3^	yield (%)^a^

1	H/4-Cl (**1b**)	H (**2a**)	2	H (**3ba**)	90
2	H/4-CH_3_ (**1c**)	H (**2a**)	2	H (**3ca**)	87
3	H/3-CH_3_ (**1d**)	H (**2a**)	2	H (**3da**)	94
4	H/4-OMe (**1e**)	H (**2a**)	1	H (**3ea**)	80^b^
5	H/4-CHO (**1f**)	H (**2a**)	4	H (**3fa**)	84
6	H/4-CO_2_Et (**1g**)	H (**2a**)	4	H (**3ga**)	75
7	H/4-CH_3_CO (**1h**)	H (**2a**)	10	H (**3ha**)	82
8	H/4-NO_2_ (**1i**)	H (**2a**)	12	H (**3ia**)	66
9	Me/4-Br (**1j**)	H (**2a**)	7	H (**3ja**)	80
10	H/H (**1k**)	H (**2a**)	4	H (**3ka**)	–^c^
11	H/4-Br (**1a**)	COOH (**2b**)	9	COOH (**3ab**)	75
12	H/4-Br (**1a**)	CH_2_OH (**2c**)	7	CH_2_OAc (**3ac**)	90
13	H/4-CH_3_ (**1c**)	CH_2_OH (**2c**)	4	CH_2_OAc (**3cc**)	94
14	Me/4-Br (**1j**)	CH_2_OH (**2c**)	7	CH_2_OAc (**3jc**)	94

^a^The reaction was run at 1 mmol scale with Amberlite 200, H^+^ form (0.35 g/mmol with respect to phenols) in air. ^b^The lower yield was partly due to crystallization to completely remove 3-(1-adamantyl)-4-methoxyphenol after chromatography. ^c^A mixture of isomers was formed.

The reaction rate was slightly decreased by electron-withdrawing groups compared to 4-bromophenol (**1a**). For example, cresols (**1c, 1d**) and 4-chlorophenol (**1b**) reacted similarly to the model reaction of 4-bromophenol (**1a**), giving the mono-adamantylation products **3ca, 3da** and **3ba** in good to excellent yields and selectivities (Table 2, entries 1–3). No isomers of **3da,** e.g., 2-(1-adamantyl)-3-methylphenol or 3-methyl-4-(1-adamantyl)phenol, were observed for m-cresol (**1d**). The reaction of phenols bearing an electron-withdrawing group, e.g., –CHO (**1f**), –COCH_3_ (**1h**), –CO_2_Et (**1g**) and –NO_2_ (**1i**), needed a longer time to afford the 2-adamantylation products in good yields (Table 2, entries 5–8). When 4-methoxyphenol (**1e**) was subjected to **2a** under similar conditions, both 2-(1-adamantyl)-4-methoxyphenol (**3ea**) and its isomer 3-(1-adamantyl)-4-methoxyphenol (**3ea’**) formed in 9:1 molar ratio as determined by ^1^H NMR analysis of the crude products, indicating that the hydroxy group (–OH) favourably directed the adamantylation compared to the methoxy group (–OMe), although the reaction of 4-bromoanisole (**1j**) provided 2-(1-adamantyl)-4-bromoanisole (**3ja**) as the solely isolated adamantylation product (Table 2, entries 4 and 9). However, phenol itself reacted to give a mixture of bi- and mono-adamantylphenol isomers, whereas 1,4-hydroquinone formed an insoluble solid material immediately upon being heated with sulfonic acid resin in acetic acid.

On the 1-adamantanol counterpart, the presence of the carboxyl acid group and the carbinol group appeared not to affect the adamantylation significantly. The reaction of 3-hydroxy-1-adamantanecarboxylic acid (**2b**) with 4-bromophenol (**1a**) gave the desired product **3ab** in 75% yield (Table 2, entry 11). Interestingly, reactions of 3-hydroxymethyl-1-adamantanol (**2c**) with 4-bromophenol (**1a**), *p*-cresol (**1c**), or 4-bromoanisole (**1j**), afforded solely acetylated adamantylation products, 2-(3-acetoxymethyl-1-adamantyl)-4-bromophenol (**3ac**), 2-(3-acetoxymethyl-1-adamantyl)-*p*-cresol (**3cc**), or 2-(3-acetoxymethyl-1-adamantyl)-4-bromoanisole (**3jc**) in 90%, 94% and 94% yields, respectively (Table 2, entries 12–14). That is, the carbinol group was completely acetylated under the reaction conditions. Moreover, no unacetylated product was detected during the monitoring of the reaction progress, implying that acetylation of carbinol proceeded faster than the adamantylation under these acidic conditions.

## Conclusion

In summary, we have developed a clean process for the synthesis of 2-adamantylphenol derivatives in acetic acid using a commercially available, strongly acidic cross-linked polystyrene-type sulfonic acid resin as a recyclable catalyst. The features of this process include (a) an almost waste-free procedure, except for the regeneration of the resin catalyst at its first use, after conversion of the sole byproduct, water, to the solvent acetic acid by anhydride; (b) the acidic ion-exchange-resin catalyst could be readily separated from the products by simple filtration and directly reused at least ten times without any reactivation, whilst showing no significant loss of activity; (c) the technique is effective with a wide range of substituted phenols giving *o*-adamantylation products with respect to the OH group, in good to excellent yields with high selectivities. The key intermediate of adapalene, 2-(1-adamantyl)-4-bromophenol, was produced by this procedure in excellent yields under the optimized conditions.

## Experimental

### General

Unless stated otherwise, all reagents and chemicals obtained commercially were used without further purification. Melting points are reported uncorrected and were recorded by using an electrothermal melting-point apparatus. ^1^H and ^13^C NMR spectra were recorded on a Bruker Avance Spectrometer. Mass-spectrum analysis was performed at the Center for Analysis, ECUST. 3-Hydroxyadamantane-1-carboxylic acid and 3-(hydroxymethyl)-1-adamantol were purchased from Alfa Aesar and Sigma-Aldrich, respectively.

### Regeneration of the sulfonic acid resins (H^+^ form)

Sulfonic acid resins (Amberlite 200 and Amberlite-IR 120, Polysciences Inc.) were regenerated following a procedure reported in the literature [28]: 100 g of resin was stirred with 20% sulfuric acid (500 mL) overnight, then filtered and washed with deionized water until the washings reached a pH value of 5–6. The resin was then washed with THF (2 × 50 mL), dried in vacuum at 90 °C for 2 h, and kept over P_2_O_5_ in a vacuum desiccator.

### Representative procedure for the acidic-ion-exchange-resin-catalyzed adamantylation with resin recycling

**2-(1-Adamantyl)-4-bromophenol (3aa)** [29]: To a suspension of dry sulfonic acid resin (Amberlite 200, H^+^ form, 5.0 g) in acetic acid (15 mL), adamantan-1-ol (**2a**; 2.43 g, 16 mmol) and *p*-bromophenol (**1a**; 2.60 g, 15 mmol) were added. The mixture was heated to 100 °C (bath temperature) and stirred for 2 h. After being cooled to about 60 °C, acetic anhydride (1.61 g, 16 mmol) in acetic acid (5 mL) was added to the reaction mixture and was stirred for 30 min followed by filtration in order to separate the resin catalyst from the solution. The resin was washed with acetic acid until it was free of product and was directly reused in the next run. The filtrate and the washings were combined, from which acetic acid was recovered by distillation, and the crude residue was purified by recrystallization from petroleum ether/CH_2_Cl_2_ to afford 2-(1-adamantanyl)-4-bromophenol (**3aa**) as a colourless fine crystals (4.48 g, 98%); mp 146–148 °C; ^1^H NMR (400 MHz, CDCl_3_, 25 °C) δ 7.29 (d, *J =* 2.8 Hz, 1H), 7.14 (dd, *J*_1_ = 8.4 Hz, *J*_2_ = 2.4 Hz, 1H), 6.52 (d, *J* = 8.4 Hz, 1H), 4.80 (s, 1H, OH), 2.08 (s, 9H), 1.77 (s, 6H); ^13^C NMR δ 153.54, 138.78, 130.24, 129.34, 118.40, 113.26, 40.30, 36.94, 28.95.

### Representative procedure for ion-exchange-resin-catalyzed adamantylation on a small scale

**2-(1-Adamantyl)-4-chlorophenol (3ba):** A mixture of adamantan-1-ol (**2a**; 0.160 g, 1.05 mmol), 4-chlorophenol (**1c**; 0.128 g, 1.0 mmol) and resin (0.35 g) was stirred at 90 °C for 2 h in acetic acid (2 mL). After the reaction was complete, the resin catalyst was filtered and washed with ethyl acetate. The solvents were removed from the combined filtrate to give the crude product, which was further purified by column chromatography (petroleum ether/ethyl acetate as eluent) to afford a white powder (0.236 g, 90%); mp 139–141 °C; ^1^H NMR (500 MHz, CDCl_3_) δ 7.15 (d, *J* = 3.5 Hz, 1H), 6.99 (dd, *J*_1_ = 3.0 Hz, *J*_2_ = 10.5 Hz, 1H), 6.53 (d, *J* = 10.5 Hz, 1H), 4.86 (s, 1H), 2.07 (s, 9H), 1.76 (s, 6H); ^13^C NMR δ 153.05, 138.30, 127.36, 126.32, 125.67, 117.87, 40.30, 36.95, 28.96; ESI–HRMS: [M − 1]^+^ calcd for C_16_H_18_OCl, 261.1052; found 261.1046.

### Characterization data for the adamantylation products

**2-(1-Adamantyl)-4-methylphenol (3ca)** [30]: mp 129–131 °C; ^1^H NMR (400 MHz, CDCl_3_, 25 °C) δ 7.06 (s, 1H), 6.90 (d, *J* = 7.6 Hz, 1H), 6.58 (d, *J* = 8.0 Hz, 1H), 4.68 (s, 1H, OH), 2.32 (s, 3H), 2.17 (s, 6H), 2.12 (s, 3H), 1.83 (s, 6H); ^13^C NMR δ 152.18, 136.17, 129.71, 127.72, 127.03, 116.68, 40.63, 37.14, 36.61, 29.12, 20.88.

**2-(1-Adamantyl)-5-methylphenol (3da)** [30]: mp 84–86 °C; ^1^H NMR (400 MHz, CDCl_3_) δ 7.14 (d, *J* = 8.0 Hz, 1H), 6.76 (d, *J* = 8.0 Hz, 1H), 6.51 (s, 1H), 4.85 (s, 1H), 2.30 (s, 3H), 2.16 (s, 6H), 2.12 (s, 3H), 1.83 (s, 6H); ^13^C NMR δ 154.25, 136.71, 133.57, 126.89, 121.48, 117.66, 40.78, 37.15, 36.39, 29.13, 20.60.

**3-(1-Adamantyl)-4-hydroxyanisole (3ea):** White powder; mp 207–209 °C; ^1^H NMR (500 MHz, CDCl_3_) δ 6.81 (s, 1H), 6.59 (br s, 2H), 4.62 (s, 1H), 3.76 (s, 3H), 2.11 (s, 6H), 2.07 (s, 3H), 1.77 (s, 6H); ^13^C NMR δ 153.57, 148.62, 137.86, 117.05, 113.95, 110.43, 55.71, 40.42, 37.04, 36.82, 29.04; ESI–HRMS: [M − 1]^+^ calcd for C_17_H_21_O_2_, 257.1542; found, 257.1538.

**3-Adamantyl-4-hydroxybenzaldehyde (3fa)** [31]: mp 217–219 °C; ^1^H NMR (400 MHz, CDCl_3_) δ 9.85 (s, 1H), 7.79 (s, 1H), 7.62 (d, *J* = 8.4 Hz, 1H), 6.79 (d, *J* = 8.0 Hz, 1H), 6.09 (s, 1H), 2.14–2.10 (m, 9H), 1.79 (s, 6H); ^13^C NMR δ (125 MHz, C_5_D_5_N) δ 191.71, 164.37, 138.12, 130.79, 129.92, 129.80, 117.89, 40.87, 37.70, 29.82.

**2-(1-Adamantyl)-4-hydroxybenzoic acid ethyl ester (3ga):** White powder; mp 250–252 °C; ^1^H NMR (500 MHz, C_6_D_6_N) δ 13.85 (s, 1H), 9.80 (d, *J* = 3.0 Hz, 1H), 9.53 (dd, *J*_1_ = 2.5 Hz, *J*_2_ = 10.5 Hz, 1H), 8.66 (d, *J* = 10.0 Hz, 1H), 5.88 (q, *J* = 9.0 Hz, 2H), 3.85 (d, *J* = 2.5, 6H), 3.54 (s, 3H), 3.25 (m, 6H), 2.78 (t, *J* = 9.0 Hz, 3H); ^13^C NMR δ 167.59, 162.83, 137.37, 129.95, 129.89, 122.07, 117.36, 61.00, 40.98, 37.71, 29.84, 15.08; ESI–HRMS: [M − 1]^+^ calcd for C_19_H_23_O_3_, 299.1647; found, 299.1648.

**3-(1-Adamantyl)-4-hydroxyacetophenone (3ha):** White powder; mp 183–185 °C; ^1^H NMR (400 MHz, CDCl_3_, 25 °C), δ 7.92 (d, *J* = 2.0 Hz, 1H), 7.74 (dd, *J*_1_ = 8.4 Hz, *J*_2_ = 2.0 Hz, 1H), 7.01 (s, 1H), 6.80 (d, *J* = 8.4 Hz, 1H), 2.60 (s, 3H), 2.16, 2.10 (s + s overlapped, 9H), 1.80 (s, 6H); ^13^C NMR δ (100 MHz, C_5_D_5_N, 25 °C) δ 197.09, 163.02, 137.39, 129.90, 129.35, 128.59, 117.18, 41.01, 37.76, 29.89, 26.78; ESI–HRMS: [M − 1]^+^ calcd for C_18_H_21_O_2_, 269.1542; found, 269.1540.

**2-(1-Adamantyl)-4-nitrophenol (3ia):** White powder; mp 227–229 °C; ^1^H NMR (500 MHz, C_6_D_6_N) δ 8.29 (d, *J* = 3.5 Hz, 1H), 8.10 (dd, *J*_1_ = 3.5 Hz, *J*_2_ = 11.0 Hz, 1H), 7.06 (d, *J* = 11.5 Hz, 1H), 2.27 (s, 6H), 2.06 (s, 3H), 1.78 (m, 6H); ^13^C NMR δ 164.76, 141.08, 138.14, 124.20, 124.16 (overlapped with C_6_D_6_N), 117.31, 40.60, 37.81, 37.57, 29.74; ESI–HRMS: [M − 1]^+^ calcd for C_16_H_18_NO_3_, 272.1287; found, 272.1291.

**2-(1-Adamantyl)-4-bromoanisole (3ja)** [29]: mp 136–138 °C; ^1^H NMR (400 MHz, CDCl_3_, 25 °C) δ 7.31 (s, 1H), 7.28 (d, *J* = 8.4 Hz, 1H), 6.75 (d, *J =* 8.8 Hz, 1H), 3.83 (s, 3H), 2.08 (s, 9H), 1.79 (s, 6H); ^13^C NMR δ 157.95, 140.83, 129.79, 129.33, 113.36, 113.32, 55.23, 40.39, 37.22, 37.06, 29.07.

**2-(3-Acetoxymethyl-1-adamantyl)-4-bromophenol (3ac):** White powder; mp 184–186 °C; ^1^H NMR (400 MHz, DMSO-*d*_6_) δ 9.65 (s, 1H), 7.16 (dd, *J*_1_ = 8.4 Hz, *J*_2_ = 2.4 Hz, 1H), 7.11 (d, *J* = 2.4 Hz, 1H), 6.73 (d, *J* = 8.4 Hz, 1H), 3.69 (s, 2H), 2.10 (s, 2H), 2.01–1.94 (m, 7H), 1.80 (s, 2H), 1.60–1.50 (m, 2H), 1.49 (m, 4H); ^13^C NMR δ 170.38, 155.38, 137.47, 129.14, 128.80, 118.32, 110.38, 72.89, 40.99, 39.04 (overlapped with DMSO-*d*_6_), 38.04, 36.62, 35.77, 33.69, 28.09, 20.57; ESI–HRMS: [M − 1]^+^ calcd for C_19_H_22_BrO_3_, 377.0752; found, 377.0756.

**2-(3-Acetoxymethyl-1-adamantyl)-4-methylphenol (3cc):** White powder; mp 217–219 °C; ^1^H NMR (400 MHz, DMSO-*d*_6_) δ 8.97 (s, 1H), 6.85 (s, 1H), 6.78 (d, *J* = 8.0 Hz, 1H), 6.63 (d, *J* = 8.0 Hz, 1H), 3.69 (s, 2H), 2.17 (s, 3H), 2.10 (s, 2H), 2.01–1.95 (m, 7H), 1.67 (s, 2H), 1.65–1.55 (m, 2H), 1.50 (br s, 4H); ^13^C NMR δ 170.46, 153.58, 134.47, 126.84, 126.75, 126.69, 116.14, 73.02, 41.38, 39.30 (overlapped with DMSO-*d*_6_), 38.20, 36.26, 35.95, 33.71, 28.19, 20.60; ESI–HRMS: [M − 1]^+^ calcd for C_20_H_25_O_3_, 313.1804; found, 313.1798.

**2-(3-Acetoxymethyl-1-adamantyl)-4-bromoanisole (3jc):** White powder; mp 110–112 °C; ^1^H NMR (400 MHz, CDCl_3_) δ 7.27 (d, *J* = 9.6 Hz, 1H), 7.26 (s, 1H), 6.73 (d, *J* = 9.2 Hz, 1H), 3.80 (s, 3H), 3.74 (s, 2H), 2.16 (s, 2H), 2.06 (s, 3H), 2.00–1.95 (m, 4H), 1.83 (s, 2H), 1.75–1.65 (m, 2H), 1.55 (br s, 4H); ^13^C NMR δ 171.32, 157.82, 139.86, 129.68, 129.56, 113.40, 113.28, 73.92, 55.21, 41.78, 39.78, 38.61, 37.44, 36.31, 34.09, 28.75, 20.91; ESI–HRMS: [M]^+^ calcd for C_20_H_25_BrO_3_, 392.0987; found, 392.0984.

**2-(3-Carboxy-1-adamantyl)-4-bromophenol (3ab):** White powder; mp 209–211 °C; ^1^H NMR (400 MHz, DMSO-*d*_6_) δ 12.02 (br s, 1H), 9.68 (br s, 1H), 7.17 (d, *J* = 8.0 Hz, 1H), 7.11 (s, 1H), 6.74 (d, *J* = 8.4 Hz, 1H), 2.11–2.01 (m, 4H), 1.95–1.92 (m, 4H), 1.80 (s, 4H), 1.66 (s, 2H); ^13^C NMR δ 178.36, 155.37, 137.28, 129.25, 128.78, 118.37, 110.42, 40.56, 40.51 (overlapped with DMSO-*d*_6_), 38.62, 37.90, 36.57, 35.41, 28.11; ESI–HRMS: [M − 1]^+^ calcd for C_17_H_18_BrO_3_, 349.0439; found, 349.0440.
